# Biomimetic Approaches for Human Arm Motion Generation: Literature Review and Future Directions

**DOI:** 10.3390/s23083912

**Published:** 2023-04-12

**Authors:** Urvish Trivedi, Dimitrios Menychtas, Redwan Alqasemi, Rajiv Dubey

**Affiliations:** 1 Department of Mechanical Engineering, University of South Florida, Tampa, FL 33620, USA; alqasemi@usf.edu (R.A.); dubey@usf.edu (R.D.); 2 Department of Physical Education & Sport Science, Democritus University of Thrace, Panepistimioupoli, 69100 Komotini, Greece; dmenycht@affil.duth.gr

**Keywords:** human motion control, biomimetics, humanoid robots, musculoskeletal model

## Abstract

In recent years, numerous studies have been conducted to analyze how humans subconsciously optimize various performance criteria while performing a particular task, which has led to the development of robots that are capable of performing tasks with a similar level of efficiency as humans. The complexity of the human body has led researchers to create a framework for robot motion planning to recreate those motions in robotic systems using various redundancy resolution methods. This study conducts a thorough analysis of the relevant literature to provide a detailed exploration of the different redundancy resolution methodologies used in motion generation for mimicking human motion. The studies are investigated and categorized according to the study methodology and various redundancy resolution methods. An examination of the literature revealed a strong trend toward formulating intrinsic strategies that govern human movement through machine learning and artificial intelligence. Subsequently, the paper critically evaluates the existing approaches and highlights their limitations. It also identifies the potential research areas that hold promise for future investigations.

## 1. Introduction

Micro and nanotechnology have been at the forefront of researchers’ investigations over the last few decades. Rapidly, scientists create new gadgets that consist of a chip that is a fraction of the size of human hair. In a laboratory setting, imagine a situation in which a nanochip begins to grow, attaches to other nanochips, and in time becomes a microchip. Eventually, it will be able to grow to the point where it will attach itself to other materials in order to re-establish its form in order to construct itself into a robot. This robot can access an unlimited amount of knowledge through wireless internet technology, which helps it grow even faster. A fictional scenario like this would appear in a Hollywood science fiction film. However, it is inspired by real-life biological cases, such as the germination of seeds or the development of a human child from an egg to an adult. Despite all the advances in machine learning and artificial intelligence, it remains challenging to mimic mother nature in many respects [1].

As a new era of post-humanization is ushered in, robots are expected to assist humans in improving their lives by living, working, and assisting with daily tasks. Robots can be successfully introduced into the human-designed environment only if they are safe, reliable, and user-friendly. Keeping this in mind, researchers are trying to develop robots with human-like structures and motion. Inspired by the idea of Bionics, Otto H. Schmitt coined the term biomimetics in 1969 [2]. The name derives from bio, meaning life, and mimesis, meaning to imitate [1]. This new subject emphasizes the research, analysis, and imitation of nature’s methods and designs. It is relatively easy to copy some of nature’s basic designs, however, re-creating some of the human-like motion using different methods of optimization is still a very challenging task. In the last decade, several remarkable developments have occurred in the field of humanoid robots. The first humanoid robot was developed in 1495 by Leonardo da Vinci, an Italian mathematician [3]. After more than 527 years, researchers from the United Kingdom developed a robotic device that resembled a human, named “Ameca” [4]. In the course of making advanced humanoid systems, several companies, including Honda [5], GM [4], Boston Dynamics [6], and research institutes such as Waseda University, Japan [7], Osaka University, Japan [8], and many others across the globe have presented their humanoid models to the research community [4]. According to Tanie et al. [9], there has been tremendous interest in using robots in various personal and social environments in the robotics community.

With the recent development of machine learning and artificial intelligence, research in the area of robotics has benefitted tremendously. The deployment of new robotic technologies is making significant strides in augmenting human-designed environments with robots from industry fields to many others, such as hospitals, homes, space exploration, and military applications [9,10]. In order to meet the requirements for the various applications, robotic systems have advanced in terms of their structural design, dexterity, manipulation, adaptability, and intelligence.

Studies have shown that a robot’s motion predictability significantly impacts the human collaborator’s performance when interacting with the manipulator [10]. Using a robotic manipulator with more human-like motions makes it easier for human co-workers to predict an arm’s motion while working together in a shared workspace, reducing anxiety and increasing risk awareness [11]. In order to achieve this sense of security, there is a need to develop robust motion planning algorithms that can successfully mimic human motion. With the advancements in artificial intelligence, they will gradually augment automation in the industry. In addition, they will serve and assist humans in shared workspaces [10].

Biomimetics and biomechanics are intimately connected fields that rely on a deep understanding of human physiology. This review investigates how motion analyses, biomimetic motion creation, and biomechanical analyses intersect with one another. Moreover, this paper presents a brief summary of the various redundancy resolution methodologies used in motion generation for mimicking human motion. By extensively analyzing the current literature, we identify promising avenues for future research and significant constraints for the existing biomimetic approach. Moreover, this review article cites other scholarly publications that offer comprehensive analyses of the diverse aspects of biomimetics, imitation learning, and learning from demonstration techniques for human arm motion control. The organization of the rest of this paper is as follows. A broader classification of biomimetics is presented in Section 2, which contains a summary of the literature review, a discussion of the various methods of data collection, and an overview of the various robot manipulators and simulation platforms that are available. The different study methodologies are the focus of Section 3, followed by Section 4, which provides detailed insights regarding the types of movement that the different redundancy resolution methods often generate. To conclude, the different research gaps and potential future work are identified and discussed in Section 5. A summary of the article is presented in Section 6.

## 2. Classification of Biomimetics Literature

**Figure 1:**
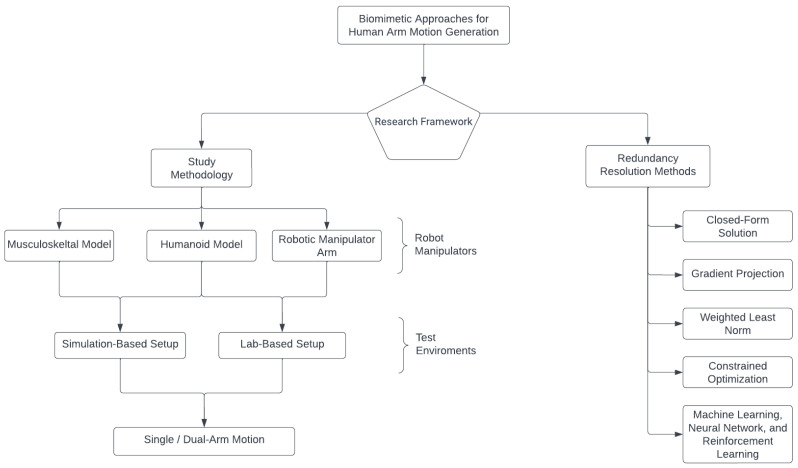
The organization flow chart of the literature review.

This literature review aims to document, categorize, and discuss the results and limitations of the various experimental setups and redundancy resolution methods that mimic human motion when performing various activities for daily living. The classification of the literature based on the experimental setup and redundancy resolution methods are presented in the chart illustrated in Figure 1.

Research groups all over the world are exploring different optimization strategies in order to improve the reliability of redundant robots in the workplace. A few literature reviews have been done in this domain. Gulletta et al. [12] presented a more elaborate discussion on the relevant principles and matrices relating to the control of human motor movements. In addition, this paper reviewed and listed the various limitations associated with human motion mimicry. A comprehensive literature review was published by Argall et al. [13] on robot learning from demonstration (Lfd). They briefly described numerous ways for collecting data and the process of deriving policies, including multi-criteria matching and dynamic modeling. A review article by Breazeal et al. [14] provided an overview of robotic imitation’s social- and task-oriented aspects. In the conclusion section of this article, the authors provided a brief overview of the limitations of imitation-inspired learning and the directions for further research. On the other hand, a significant amount of work occurred in human arm movement control, particularly in computational theories. A brief review of the descriptive and dynamic models for human movement control was presented by Campos et al. [15]. This paper provided an overview of control and computational modeling and their potential relevance for rehabilitation.

Section 2.1 presents a brief overview of the literature review outline. Section 2.2 addresses the various methods for recording human demonstrations, while Section 2.3 provides a comprehensive description of the latest types of robot manipulators and their associated simulation software.

### 2.1. Outline of the Literature Review

Redundant manipulators consist of a number of joints that is greater than the number of degrees of freedom (DOF) that are required to complete a task. Over the last several years, redundant robots have captivated the robotics community due to their potential to solve a variety of problems dexterously while satisfying additional criteria. Multiple methods to control them have been the focus of much research, such as tracking joints and task spaces, maximizing performance criteria, null space optimization, etc. The primary objective of the study was to conduct a comprehensive and systematic review of the current literature on human motion mimicking and robotics, with a specific emphasis on peer-reviewed publications. To achieve this objective, the study employed various search strategies, such as keyword searches with specific search terms, including biomimetics, biomimicry, human motion generation using machine learning, imitation learning for motion generation, redundant manipulators, and redundancy resolution methods for biomimicry. The focus was on identifying articles that were published in peer-reviewed conferences or journals, while excluding unpublished or non-peer-reviewed manuscripts, book chapters, and abstracts from the analysis. The search sources included the Association for the IEEEXplore, Springer, the Journal of Biomechanics and Cognitive Neuroscience, the Computing Machinery (ACM) Digital Library, MDPI, Elsevier, John Wiley & Sons, the Journal of Intelligent and Robotic Systems, and the Web of Science.

The study scrutinized and discussed the dependability, efficiency, advantages, and limitations of each approach for generating human-like arm motion, with particular emphasis on the redundancy resolution methods, simulation software, and robotic manipulators employed for experimentation. Initially, the search yielded 128 publications, which were potentially relevant to the study. However, after applying the inclusion criteria, only 85 publications were deemed eligible for inclusion in the review. The included publications, representing the most current research on the topic, were published after 2003. While some publications from the 1980s and 1990s included in the review described the methodology that later researchers implemented in the latest publications on redundant manipulators.

**Figure 2:**
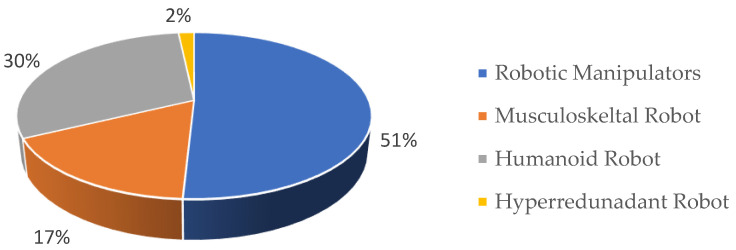
Percentage of papers describing the different robotic systems.

The percentage of papers describing the various robotic systems is illustrated in Figure 2. Based on our literature review, the overall research comprised approx. 51% robotic manipulators, 30% humanoid robots, 17% musculoskeletal robots, and 2% hyper-redundant robots. It was evident from the literature that humanoids and seven degree of freedom manipulators served as the ideal platforms for testing biomimetic algorithms due to their high degree of anthropomorphism.

**Figure 3:**
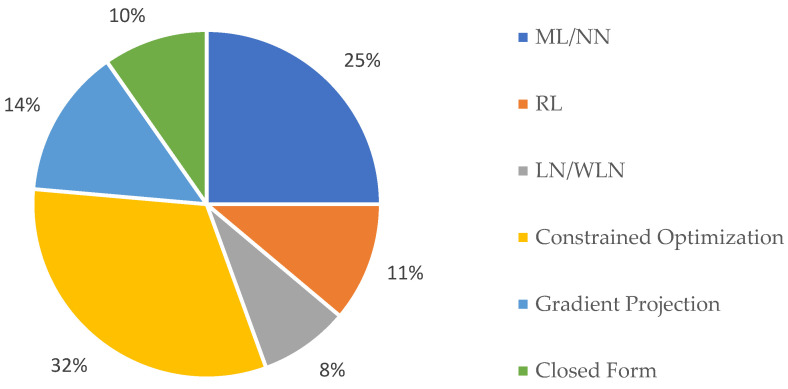
Percentage of research articles describing the redundancy resolution methods.

A redundant manipulator chooses a configuration among an infinite number of other configurations based on the performance criteria. This ensures a higher dexterity and flexibility in the motion planning. The human upper body is a highly redundant structure capable of executing various tasks with a high agility and with a relatively large number of degrees of freedom, typically ranging from 20 to 25 DOF [16]. As a result, solving the inverse kinematics (IK) becomes challenging. Many researchers have tried different approaches to simulate human-like motion in robots. The pie chart in Figure 3 shows the percentage of the research articles in diverse categories describing the various methods that were used on the different robotic systems. Overall, 32% of the papers employed a constrained optimization, while 14% used gradient projection algorithms. When reviewing the latest research literature, it was evident that there was a strong trend toward neural networks/machine learning (ML/NN) research (25%) and reinforcement learning (RL) research (11%), with only 8% of the research focusing on the least-norm and weighted least-norm methods (LN/WLN).

**Figure 4:**
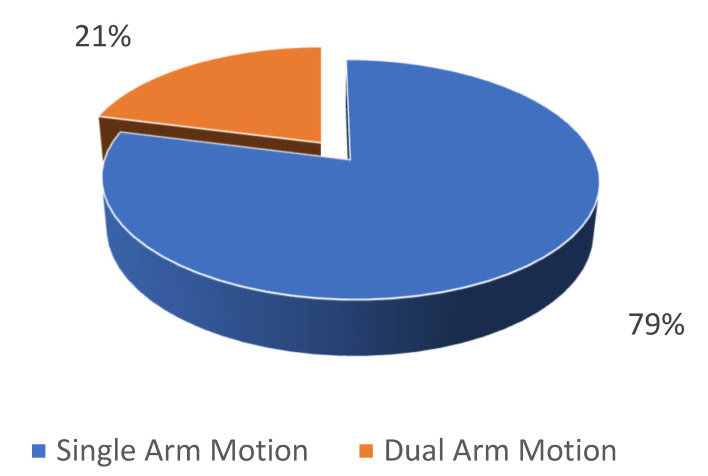
Comparative analysis of the biomimetics approach on single vs. dual robotic arms.

A redundancy resolution problem becomes more complex when two arms work together since more degrees of freedom are involved [17]. As a result, the planning motion for dual-arm robots becomes much more complex than simply mirroring the results from one arm to the other. Figure 4 illustrates the comparative analysis between the papers describing the biomimetics approach tested on single and dual robotic arm manipulators.

**Figure 5:**
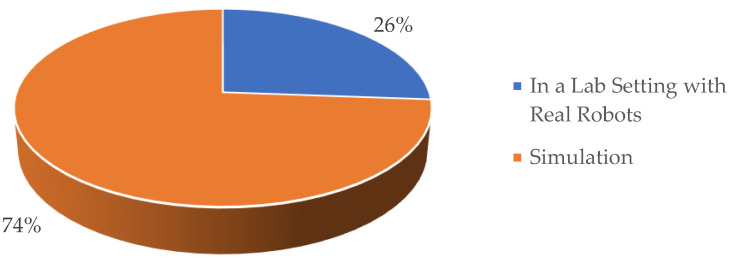
Categorization of the studies based on the experimental setup.

Figure 5 depicts a categorization of the studies that were included in the analysis based on the experimental configurations that were employed for executing the biomimetic principles on robotic systems. As seen in the figure, 74% of the included studies proposed experimental simulation setups for performing biomimetics on robot systems. As a comparison, only 26% of the studies conducted experiments on robots in a controlled lab environment.

### 2.2. Data Collection Methods

**Figure 6:**
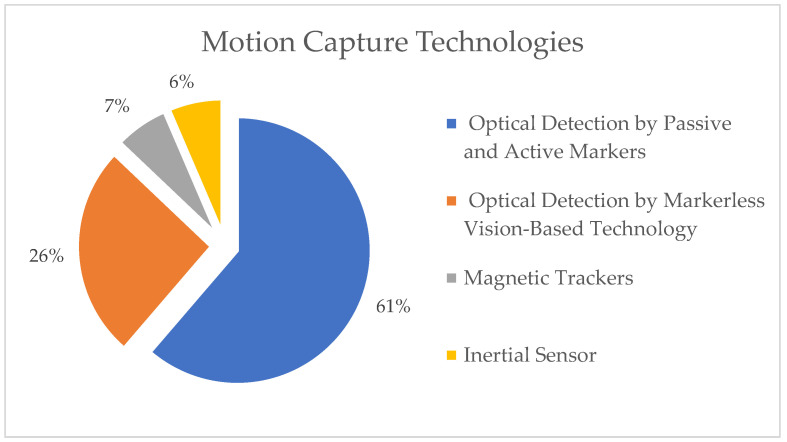
Classification of the studies based on the motion tracking technologies.

The biomimetic approach to human motor control emphasizes the importance of evaluating robot motion in the context of human motion as a benchmark [12]. The multimodal data of the joint angles, end-effector trajectory, electromyographic (EMG) signals, electroencephalography (EEG) signals, and force plate signals are all essential measurements that could be conducted to study human behavior and the performance criteria behind them. Motion tracking technologies have become more accurate in recent years due to increased computing power, improved algorithms, and post-processing techniques. In the market, a wide range of motion tracking technologies are available, all based on their physical principles and designed to track the position and orientation of points of interest. A classification of the included research studies based on motion tracking technologies is shown in Figure 6.

One of the most widely used technologies for capturing motion is the optical motion capture system, which uses passive and active markers due to its real-time tracking accuracy [18]. Approximately 61% of the total literature reviewed in this paper on biomimetics used optical tracking with passive and active markers to measure human subject motion. Infrared cameras are placed around the subject to triangulate a marker position on the different body parts using 10 to 12 fixed high-speed and high-resolution cameras. An infrared camera’s frequency for capturing high-contrast images varies from 25 Hz to 2 kHz, depending on the number and type of markers [19]. To avoid errors caused by occlusion, most technologies utilize a minimum of three cameras to track the markers [20]. In contrast to the active markers, the passive markers are not constrained by wires or battery packs. However, the downside is that each marker position must be corrected using various post-processing techniques.

Optical tracking systems provide the most accurate measurement for the 3D position of the markers since active markers, or reflective emitting diodes (IREDs), have different frequencies and are easy to distinguish from one another. However, they require minor post-processing. The significant drawbacks of this device are the required size of its workspace and its portability.

Markerless motion capture systems based on RGB cameras and infrared depth sensors are a research area that is currently being explored. A large number of research groups have used three RGB cameras to study the biomechanics of the human body by tracking QR code-based markers placed on the subject’s body [21]. The system’s accuracy is reasonable when using the RGB cameras if the motions are limited to a 2D space. In contrast, the Kinect cameras offered by Microsoft provide a better accuracy when measuring motion in 3D space [22]. A Kinect camera consists of an infrared light projector, an IR camera, and an RGB video camera. As long as the resolution of the video output is high enough, it can track the whole body at a frequency between 9 Hz and 30 Hz, providing detailed information regarding the position of the joints. In order to ensure the reliability and accuracy, technical advancements in software and hardware development are necessary to ensure that markerless motion tracking systems can achieve a higher accuracy level than marker-based motion tracking systems.

One of the technologies available for determining human motion is inertial sensors that are composed of a triaxial accelerometer and a gyroscope for measuring the acceleration and rotational velocity [23]. Each sensor can be strategically placed in such a way that each segment is oriented accurately by its position. The Xsens Moven motion capturing suit is capable of recording 3D kinematics of the entire human body by adjusting the location of the sensors, adjusting the Earth’s magnetic field, and calibrating the subject dimensions at a rate of 100 Hz with a precision of 1° RMS. In order to gain a better understanding of the kinematic motion of the joints using the inertial sensors, researchers should read the work by Jung et al. [24].

Liarokapis et al. [25] used magnetic markers in order to record upper limb motion. Triaxial transmitters measure each axis’ magnetic field sequentially by generating DC pulses. The trackers measure the three-dimensional position and orientation of a magnetic tracking system. A magnetic tracking system has the advantage of not introducing errors due to occlusion, allowing the markers to be placed anywhere on the subject. A magnetic tracking system’s portability has the advantage of using an optical tracking system. The only disadvantage of using this system is its sensitivity toward metallic objects. Table 1 catalogs the significant highlights of each type of motion capture system.

### 2.3. Robot Manipulators and Simulation Softwares

The advent of post-humanization has resulted in researchers developing robots by adapting mechanisms and abilities that are inspired by nature and studying scientific approaches to understand the natural principles in order to develop a more realistic robotic system. This section provides an overview of the different experimental setups used in the selected biomimetics research studies, and Figure 2 and Figure 3 illustrate the percentage of papers employing the various robotic systems and their classifications based on the various redundancy resolution methods used in the different robotic systems.

The biped humanoid robot MAHRU II was designed and developed by Samsung Electronics Co., Ltd. with the Korean Institute of Science and Technology (KIST) in 2006 [26]. In comparison to other humanoid robots, MAHRU II is a network-based humanoid robot with a total of 33 DOFs, out of which, 15 DOFs are in the upper limbs (one DOF in the waist, seven DOFs in each arm). MAHRU III is an updated version with a total of 44 DOFs. The Fujitsu HOAP-3 humanoid robot has a height of 60 cm and a weight of 8 kg [27]. The robot has 28 DOFs. It has six DOFs in each leg, five DOFs in each arm, one DOF in the waist, one DOF in each hand, and three DOFs in the chest. Two charge-coupled device (CCD) cameras are mounted on the head, as well as microphones, speakers, and force sensors to measure the grasping force. The iCub [28] has been designed to perform object manipulation and mobility. In total, 30 DOFs are allocated to the upper part of the body. Each hand has nine DOFs with three independent fingers. Each arm has seven DOFs and each leg has six DOFs. The iCub weighs approximately 22 kg. In addition, it is equipped with two RGB cameras, a microphone, a speaker, and force sensors. ROMEO is a 1.4-m-tall humanoid robot with an approximate weight of 40 kg [29]. In addition to its 37 DOFs, it has two DOFs in each eye, seven DOFs in each arm, one in each foot, six in each leg, and three DOFs in the backbone. The primary purpose of developing this robot was to assist elders and people with disabilities in their daily activities. The NAO [30,31] robot is a humanoid robot with a total of 25 DOFs, 11 degrees for the lower part of the body and 14 degrees for the upper part, which includes the trunk, the arm, and the head. In addition to the two RGB cameras, two sonar sensors, a microphone, a speaker, and tactile/force sensors, each arm contains two DOFs in the shoulder, two in the elbow, and one in the wrist.

To emulate the human arm configuration, many research groups [32,33] have designed a mobile platform consisting of two industrial robotic arms UR5 with six DOFs, where each arm is equipped with 16 DOFs in an Allegro Hand. Similarly, ABB's Friendly Robot for Industrial Dual-Arm Assembly (FRIDA) is a dual-arm robot with seven degrees of freedom in each arm. [34]. Some research groups have tested their human mimicking algorithm on seven-DOFs anthropomorphic industrial manipulators, such as the PA-10 from Mitsubishi [25,34]. At the same time, some research groups have designed their own seven-DOFs anthropomorphic manipulator [35]. These manipulators are equipped with torque and force sensors to measure the external forces and joint moments while performing different tasks.

Parallel to the advancement and evolution of robotics research and modeling, virtual robotic simulation also has a long and vivid history. In the early 1980s, today’s Dassault system released the early version of its graphic simulation software named IGRIP in competition with the ROBOCAD product developed by Technomatrix [36]. Both of these software impressed users with their visualization. However, due to a lack of processing power, this simulation software could not predict robot motion, making them unreliable for research purposes [36]. After four decades of development, present-day simulation software such as MATLAB, OpenSim, Robot Operating System (ROS), ADAMS [37] and Multi-joint dynamic with Contact (Mujoco), provide modeling, programming, refinement, and graphic animation all in one place [38]. In a study by Kim et al. [39], a comparative analysis of two musculoskeletal modeling systems, OpenSim and AnyBody, was presented. The authors evaluated the differences and similarities between the two systems in terms of their model structures, mathematical formulations, and computational efficiencies. These resources allowed researchers to experiment in a variety of robots without the need to access to the physical mechanism. More importantly, it made it possible to develop prototypes for more complex systems and work on their control algorithms before deciding whether they were worth building.

## 3. Research Framework

This section presents a comprehensive survey of the study methodology and the different redundancy resolution techniques utilized in the domain of robot motion planning with the objective of emulating human movements.

### 3.1. Study Methodolgy

Biomimetic research aims to develop robots and software platforms that can replicate human movements in a human-centric environment. The simulation involves the creation of a model of a physical system based on a theoretical model, followed by the implementation and analysis of the model implementation [38]. With increasing robot complexity, the simulation becomes more crucial for the motion optimization and verification before the algorithms are implemented in the physical robots. Based on Figure 5, 72% of the studies in biomimetics employed simulation platforms, while 28% utilized actual robots. This outcome was influenced by the experiment costs, availability of robotic devices, and the tasks to be performed. Silva et al. [40] demonstrated that the human–robot collaboration was facilitated by anthropomorphic robots that moved like humans. Such characteristics enable a more efficient and natural human–robot interaction, allowing the human user to understand the robot’s movements more clearly as goal-directed actions.

Developing autonomous object manipulation capabilities in anthropomorphic robots is a very challenging problem since there are many factors to consider.

1. A vital attribute of these robots is that they have a high number of degrees of freedom (DOFs). Redundancy provides flexibility and the capability to compensate for lost control as fast as possible and adapt to new dynamics. However, in cognitive robotics, simultaneously controlling multiple degrees of freedom in a predictive/purposeful manner can be computationally challenging.
2. Generating of an optimum end-effector trajectory can avoid collisions between the self and the objects in the environment.
3. Optimizing the various performance criteria can generate joint angle trajectory paths that mimic human movements.

### 3.2. Robot Manipulators

Throughout the past few decades, many robots have been developed and put into research to mimic human anthropomorphism. Musculoskeletal robots strive to exhibit both a degree of compliance and a fairly natural distribution and dynamics of the mass to mimic human mechanical properties. Among the many applications of musculoskeletal robots, particularly the ability to easily control the skeletal muscles, one of the most significant advantages is that their actuators are lightweight and have a low inertia. Richter et al. [41] presented a design that mimicked a human arm. In this article a large amount of muscle mass was firmly attached to the torso, while the tendons, composed of very little weight, connected the muscles to the bones of the distal part of the body. Another essential factor for controlling the musculoskeletal model is the control strategy associated with the model. How humans control their bodies is the most versatile and energy-efficient way to use musculoskeletal systems. Research organizations worldwide have been working on robotic musculoskeletal systems, either on their mechanical design or on their simulation software, to help imitate human performance. Researchers at Stanford University developed a model of the upper extremity that represents the shoulder, elbow, forearm, wrist, thumb, and index finger, as well as 50 muscle compartments crossing these joints, in order to represent 15 degrees of freedom [42]. These models were also used to estimate the muscle forces and joint moments based on a muscle activation pattern. The model’s accuracy was evaluated with the maximum moment-generating capacity of each muscle group. Fan et al. [43] also applied the same approach, in which an artificial muscle-driven muscle–skeleton robotic arm based on the principles of mechanical muscles was proposed. In their work, the authors used multifilament muscles in the form of actuators that shared similar physiological characteristics with human arm muscles. These multifilament muscles were then modeled in a simulation environment, resulting in humanoid robot arm models that could be used for testing human motion. A reinforcement learning method was adopted to train the control policies to verify the movement capability of the different muscle configuration models and determine whether these models were controllable and mimicked human motion.

In the paper by Al Borno et al. [44], the authors discussed how muscle synergy models were implemented in a biomechanically realistic upper extremity model of the human body based on [42], which was designed to simulate upper extremity movements in real time. The researchers conducted several computational experiments to determine whether the synergies resulted in performance deficits during the tasks, facilitated participant movement learning, and were generalizable to other movements. In the same direction of research, Lenarcic et al. [45] also aimed to recreate well-known features of the movements of the upper extremities in three dimensions using a biomechanically realistic computational model based on [42] that reproduced the well-known features of those movements based on Fitts’ law. Sapio et al. [46] examined the muscular effort required for the positioning tasks as a method of predicting the arm configuration during reaching movements. This article proposed the use of the musculoskeletal model of Holzbaur et al. [42] to account for the kinematic coupling between the constituents of the human shoulder.

In studying human-like movement, mathematical models of or parts of the human body provide critical tools for analyzing the kinematics and dynamics associated with those movements. It is necessary to define the arm to develop a mathematical model of it. A wide range of research groups defined the arm in various ways. Lenarcic et al. [45] described it as a serial mechanism of four parts: the shoulder girdle, the upper arm, the forearm, and the hand. Chan et al. [47] proposed a model with eight DOFs and three segments: the upper arm, forearm, and hand. The model developed by Gams et al. [48] consisted of four segments: the shoulder girdle, upper arm, forearm, and hand, each of which had ten degrees of freedom. The most common method of representing the human arm is through modeling each arm as a set of seven DOFs kinematic chains, as presented in [49], with three rotational joints for the shoulder, one rotational joint for the elbow, one rotational joint for the forearm, and two rotational joints for the wrist.

A number of humanoid models that are of a similar size to that of a human are composed of several degrees of freedom (DOFs), for example, at least seven DOFs per arm, three DOFs for the torso, and six DOFs for the legs. Researchers are developing robot models that closely mimic human size, joint limits, and other kinematic parameters to mimic a human more accurately. Using the kinematic model of the ROMEO robot as a starting point, researchers from the University of Belgrade Tomic et al. [29] designed a 35-DOFs human body model that contained seven DOFs for each arm, six DOFs for each leg, one DOF for each foot, two DOFs for the neck, two DOFs for the head, and one DOF for the spine. The reference papers [4,27,30,31] also used similar robot platforms, Simon, NAO, and the HOAP-3 robot platforms. The HOAP-3 and NAO both had a total of 25 degrees of freedom. HOAP-3 had one DOF on the torso, five DOFs on each arm, four DOFs on each hand, two DOFs on each ear, and three DOFs on the neck. The NAO had 11 DOFs in its lower body, which included the legs and pelvis, and 14 DOFs in its upper body, which included the trunk and arms, as well as the head. Each leg had two DOFs at the ankle, knee, and hip. Unlike Simon’s robot platform, which had five DOFs, each arm, six legs, and two muscles on the neck were controllable. In contrast, the researchers at the University of South Florida created a kinematic robotic human body model (RHBM) to simulate the optimum upper body movement of healthy users and users with transradial prosthetics [50,51].

When it comes to redundant manipulators, there are be infinite joint angle configurations that can be used to enable an end-effector to reach a particular objective. For human-like motion generation, it would not be feasible to map the human motion directly onto the robot arm due to the different lengths and joint limitations between a human and a robot arm. Various researchers have tackled the correspondence problem using various strategies based on optimization schemes. Despite having a limited workspace, several research groups opted for six DOFs robotic arms, such as the UR5 and Jaco2 [32,52]. The UR5 arm has two DOFs in the shoulder, one DOF in the elbow, and three DOFs in the wrist. The Jaco2 arm consists of a shoulder joint with one DOF, one DOF in the elbow, three DOFs in the wrist, and one DOF in the last degree of rotation around the axis that serves to control the robotic arm. Research groups are increasingly turning to robotic arms with seven DOFs, such as the KUKA [53] and the PA-10 [54] by Mitsubishi, to improve the workspace and take full advantage of the arm redundancy. Considering a higher degree of freedom for a better redundancy resolution, the use of hyper-redundant robot arms has been an active research topic for several decades. However, their applications remain limited. Liarokapis et al. [25] demonstrated a constrained optimization strategy for hyper-redundant robot arms. The authors created robot arms with nine, eight, twenty, and twenty-one degrees of freedom that were less, equal, or greater than the average length of a human arm.

### 3.3. Test Enviroments

For the last couple of decades, a large number of simulation tools for robotic systems have been available in the market. Numerous research groups and industrial fields use these tools extensively to design control algorithms. As far as simulation tools are concerned, most of them are primarily focused on the movement of a manipulator in various environments rather than its graphical interface. All the simulation systems include kinematic or dynamic models of the robot manipulators in order to simulate the motions. There are two main types of simulation tools used in biomimetics research: (1) simulation tools that are developed on top of existing simulation systems, such as MATLAB [55], OpenSim [56], Cosimir [57] etc., and (2) tools that are designed explicitly by research groups to validate their algorithm’s performance [38].

In the study of biomechanical movement, computer models have become increasingly important. An important driver of this interest is the belief that modeling can greatly improve our understanding of how the neuromuscular and musculoskeletal systems interact to generate movement. The OpenSim software application is an open source tool that researchers use to develop musculoskeletal structures and create dynamic simulations of various movements [56]. These musculoskeletal models help study the biomechanics of different movements and neuromuscular control. These models should provide accurate representations of the muscles and joints and describe the essential interactions between them. Keeping the importance of model-accurate representations, Fox et al. [58] developed a model of the upper extremity that included 15 degrees of freedom. Researchers have used this musculoskeletal model to study human motion. Furthermore, to perform biomimetics, researchers hypothesized that the central nervous system (CNS) uses the power of modularity to simplify the control of the complex multi-dimensional musculoskeletal system by minimizing the motor commands to a small group of modules called muscle synergies, which are coupled together as a system and can be used to further analyze motion. A biomechanically realistic upper extremity model based on OpenSim was used by Al Borno et al. [44] to simulate the muscle synergies. Fan et al. [43] also used the same model to conduct reinforcement learning experiments, and Sapio et al. [46] implemented these experiments in order to optimize the muscle effort criteria.

The MATLAB software has become one of the most widely used platforms for developing models and simulations of various kinds of systems. The fundamental data types of MATLAB, the vector, and the matrix have also made the program extremely applicable to the simulation of robots and their systems [59]. Many research articles on biomimetics have referred to the MATLAB robotics toolboxes by Peter Cork [55] as the most widely used MATLAB extension. This toolbox has played an important role in designing a human-like model and programming various optimization algorithms. According to the data that were collected during the ROM tasks [60], constructed a model based on the Denavit and Hartenburg convention. The values of the various parameters of an individual’s upper body were determined by the location of their joint centers and the degrees of freedom of each joint. In contrast, Menychtas et al. [49] created a robotic model of the human body using Lie algebra. The ROM data extracted the parameters, such as the segment lengths and joint centers, to create the robot model and calculate the joint angles to analyze the various ADL tasks. The MATLAB-based robotics toolbox by Peter Cork was also used by [61] to create a kinematic model of the human arm with seven degrees of freedom. This model allowed for the analysis of the swivel motion and manipulability during the task manipulation, which could be used in order to implement human-like behavior within the humanoid robot.

Researchers have also adopted software such as the ROS [62] and Mujoco [63]. Both of these open source programs are designed to allow researchers to conduct research in robotics, biomechanics, and machine learning [38]. In addition to the fact that both of these software are open source platforms, they can also be easily integrated and controlled using a wide variety of hardware and software components, which is one of their significant advantages in robotics. In addition to interacting with the sensors, actuators, and other hardware components, the ROS and Mujoco provide several tools and libraries that facilitate communication between the different hardware components. By integrating and configuring the appropriate components, the developers can build complex robotics systems much faster, rather than constructing everything from scratch, making it much easier to build complex robotics systems. Due to their vast libraries and free online support, both software packages have become reliable tools for many researchers [43].

The demand for robot assistance has been increasing for more than two decades in various fields that share a workspace with humans, such as industry, service robotics, and rehabilitation. Simulation software offer highly reliable and cost-effective approaches to motion planning. However, robot behavior in real-world scenarios must be predictable and socially acceptable for the system to succeed. Various research groups have conducted several studies to improve the assistance quality offered by robot manipulators and humanoid robots in various real-world scenarios [64,65,66]. An article by Su et al. [67] evaluated the ability of a human to manage redundancy while controlling anthropomorphic robot arms (KUKA) during teleoperation. In order to achieve an anthropomorphic arm posture during the teleoperation tasks, they implemented a nonlinear regression method based on neural networks to generate a relationship between the elbow swivel angle of the human arm and the target hand pose. A simulation was conducted to assess the method, and lab experiments were conducted. Similar studies presented by Liu et al. [68] and Artemiadis et al. [69] also conducted a human demonstration tracking experiment to assess the algorithm efficacy of generating human-like motion by optimizing an elbow swivel angle. They employed a KUKA and a PA-10 to simulate the human motions. In contrast, Kim et al. [70] developed a method for modifying and scaling the wrist trajectory and elbow elevation angle to reproduce human-like arm motions.

An article by Chen et al. [71] presented a computational framework that leveraged the hierarchical principle of human motor control to establish hand configuration trajectories. The authors utilized a second-order kinematic model to capture the dynamic behavior of the hand and produce an external representation of the high-level motor control system. To establish the efficacy of the proposed approach, the authors conducted several highly self-reaching movements on a seven-degree-of-freedom (DOFs) anthropomorphic arm. They compared the configuration trajectory generated by the proposed method with actual human movement in a spatiotemporal profile to validate the approach’s effectiveness. Similarly, Elbasiony et al. [31] proposed an innovative interface for conveying whole body human demonstrations to robots, with a significant emphasis on integrating the learning framework and the demonstration interface. This integration enabled the NAO robot to independently learn and replicate the demonstrated motions instead of merely imitating them. To reduce the motion’s dimensionality, the authors employed dynamic time warping (DTW) for the signal alignment and utilized a Gaussian process latent variable model (GPLVM) for the modeling and replication of the demonstrated motions. Albrecht et al. [28] proposed a framework that can represent and generate motion based on the optimization of both the physically inspired principles and the analysis of human motions recorded in an unconstrained observation setting. To determine the best cost function for describing the movement of the human arm, the authors analyzed the motion recording data using a bilevel optimization approach. This cost function generated the optimal trajectory paths for a humanoid robot known as iCub that performed a similar function to a human.

### 3.4. Single/Dual-Arm Motion

As a result of recent efforts in the field of biomimetics and technological advancements in the field of robot design, dual-arm movements and manipulators with human-like characteristics are among the most trending research topics today. Based on the findings shown in Figure 4, a significant proportion of the included studies (i.e., 79%) primarily focused on proposing techniques for generating single-arm motion. Conversely, a relatively smaller percentage of studies (i.e., 21%) proposed dual-arm motion generation techniques. Several factors can be accountable for a particular outcome. Some of these factors were the manipulators and simulator platforms used for the experiments, as well as the type of tasks the experiments were designed to accomplish. The article by Zanchettin et al. [72] proposed an easy-to-follow relationship between the hand posture (position and orientation) and the swivel angle when performing a task based on the simple relationship between the hand pose and the swivel angle. Using kinematic variables, the authors concluded that this relationship could be easily applied to the control of robotic manipulators through the use of kinematic variables to explain the motion of an arm. Using volunteers to perform the simple assembly-like tasks as the subject of their experiment, the authors conducted an extensive experiment to analyze and establish the relationship between the hand poses and swivel angles using a combination of clustering and multivariate correlation statistics. Based on the results of this study, the model was successfully applied to a humanoid robot, the ABB FRIDA, and demonstrated the model’s ability to generate dual-arm movements that were human-like.

Lamperti et al. [73], proposed a comparable strategy for addressing the redundancy in an anthropomorphic dual-arm robotic system, wherein the muscular effort was minimized. The authors hypothesized that the right-hand posing problem could be resolved by minimizing the muscular activation (i.e., muscular effort) necessary to maintain the manipulator’s position against gravity. This could be achieved by computing the optimal swivel angle based on the hand position. Similarly, the left arm posture definition could be determined by identifying a left swivel angle that minimizes the muscular effort required to move the left arm. Through the utilization of the data samples obtained for the different dual-arm tasks, the authors mapped the arm configurations to eight redundant variables that defined the hand pose and swivel angle.

A paper in the same field by Garcia et al. [33] examined how a mobile anthropomorphic dual-arm robot can obtain human-like motion by studying how a mobile robot base affects the dual-arm synergies of the robot. Their work aimed to develop a human-like coordination between the robot’s translational and arm movements when performing dual-arm manipulation tasks on a table. The authors divided the paper into three phases. The initial stage of the research methodology entailed the acquisition of the human movements and their subsequent translation into the robotic system as a human operator executed manipulation tasks while approaching a table. In the subsequent phase, the acquired movements underwent analysis to establish the association between the position and orientation of the robot, the configuration of its arms, and the recorded movements. Based on the derived robot configurations, the fluctuations in these associations were scrutinized, and the dual-arm synergies were calculated from the mapped robot configurations. As a result of the computations of the synergies and correlations observed in the examined data, the Cartesian space was discretized and divided into various regions. A motion planning algorithm based on rapidly-exploring random trees (RRT) was presented in the last phase that exploited the synergies between the different regions of the Cartesian space to produce coordinated movements similar to humans.

The methodology employed by Shin et al. [74] to establish a human likeness in the models involved the utilization of specific criteria, namely the hand trajectory and elbow trajectory. These trajectories were generated using the elbow elevation angle (EEA). To this end, the study authors proposed a control method premised on the virtual dynamics model (VDM). A VDM-based controller filters the target trajectory of a motion (or force) to be controlled and then passes it on to the robot motor controller, which then executes it on the robot. The VDM-based controller enabled the robot arm to be controlled directly without needing to solve the inverse kinematics or avoid the geometrical singularities. The efficacy of the VDM-based controller was determined by comparing the arm motion generated by the proposed controller to the captured arm motion data. An object manipulation task was demonstrated using a humanoid robot to represent reaching for, grasping, and moving an object within a sequence of events. Due to its dependence on the databases, the VDM-based controller approaches did not allow for the generalization of the manipulation tasks.

A majority of the studies focused on developing techniques for generating single-arm reaching movements that imitated human-like motion, with a limited exploration of the complex manipulation movements. However, recent progress in biomimetic research facilitated the development of more intricate robotic applications, including dual-arm manipulation that leverages actual human biological and psychological patterns.

## 4. Redundancy Resolution Methods

In general, six DOFs are sufficient to place the end-effector at any point and with any orientation within the workspace. However, only a single set of joint angles that can lead to a specific position and orientation can be achieved. This mapping works well for robots working within a structured environment and performing repetitive tasks of a similar nature. However, once obstacles are introduced into the environment, and the manipulator has to perform more complex tasks, this joint-to-space mapping becomes much less dependable. The solution to this is adding redundant joints to create multiple configurations that lead to a specific position. The drawback is that more complex control algorithms are required in redundant manipulators to take advantage of the extra DOFs.

The human body is capable of performing highly complex tasks in unstructured environments, due to its numerous joints and the extremely refined control it can provide via the central nervous system. A secondary objective of biomimetic robots is to simulate human motion or, more specifically, to determine what aspects of human motion should be duplicated while maintaining a translational and rotational speed consistent with the desired end-effector (i.e., the hand). This is a task-driven approach that has allowed the problem of human motion resolution to be tackled from different angles with varying results. The purpose of this section is to review the approaches that have been attempted and to examine the scope of each method used (analyzing human motion, developing human-like control algorithms, etc.) to determine whether the results are acceptable. This is important because human motion is still an unsolved problem, and the scope of the application dictates whether the accuracy of the imitation is sufficient.

### 4.1. Closed-Form Solutions

The first family of biomimetic methods is the closed-form solution of human motion. Closed-form solutions present analytical methods to solve inverse kinematics efficiently. These methods map the end-effector’s location to a set of joints based on the robot’s geometry. Closed-form solutions are specific to each robot, but they can resolve the motion at any configuration without fail. Conceptually, they are the most direct solutions for a robot. However, redundant robots lack the one-to-one mapping between the set of joint angles and the set of end-effector Cartesian coordinates. In order to deliver a human-like configuration, additional constraints are required to be imposed on the robot’s mechanism and the tasks it performs. For instance, Liu et al. [68] used demonstrators to record upper extremity motions in order to create the key positions of the shoulder, elbow, and wrist within in a plane. The inverse kinematics solution was constrained to this plane. In essence, the robot had to reach the given end point by keeping the elbow angle on the defined plane. The method’s performance was evaluated by comparing it to real-time data from a depth camera (Kinect). Kim et al. [74] used the elbow’s elevation angle (swivel) to create a closed-form solution. To do that, they used a response surface methodology (RSM) on the motion capture data to approximate the elbow elevation angle during the motion. This restriction allowed for a humanoid robot (MAHRU, developed by the Korean Institute of Science and Technology—KIST), to imitate human-like arm motions. Correspondingly, Zanchetin et al. [75] used the swivel angle to establish a relationship between the hand and elbow to identify a kinematic constraint that led to human-like movements.

Similarly, Wang et al. [34] used the swivel angle to find a closed-form solution. A different approach was used by Ding et al. [76]. In that work, the authors used an intermediate step between the task and the joint space, which was parametrized by a triangle between the shoulder, elbow, and wrist of the human arm (the human arm triangle). In order to solve the inverse kinematics problem in a closed-form manner, the motions from the task space had to be mapped onto the human arm triangle, which then enforced the constraints that would result in an efficient solution to the inverse kinematics problem.

Another area that requires accurate recreation of human motion is that of the exoskeleton. Again, the swivel angle was used as a constraint by Kim et al. [77], and the motions were then selected based on the projection of the maximum manipulability. Later, Kim et al. [61] used a joint angle availability function to solve the inverse kinematics while accounting for the swivel angle in order to minimize the energy exchange between the user and the exoskeleton. More abstract conditions have been proposed by Chen et al. [71]. Chen and colleagues proposed using a composite force field to generate the trajectory and then used bio-inspired coefficients to find the joint angles. Even though the optimization methods need to be applied to the motion capture (MoCap) data to calculate the coefficients, the actual algorithm does not rely on any such methods, so it maintains the speed of a closed-form solution.

### 4.2. Gradient Projection Methods

The second family of solutions is the gradient projection method. These numerical methods usually resolve motion in the null space at the velocity level. This allows the extra degrees of freedom to satisfy the additional criteria without affecting the end-effector accuracy. While closed-form solutions look at the geometrical/anatomical constraints, gradient projection methods can have abstract criteria that govern motion. Their usual form is shown in Equation (1).

(1) $$\dot{q}=J^{\#}\dot{X}+\lambda (Ι-J^{\#}J)\nabla \kappa$$

where $\dot{q}$ is the joint angle velocity vector, $J^{\#}$ is a generalized inverse of the Jacobian matrix of the system (usually the Moore–Penrose inverse), λ is a scaling factor, I is the identity matrix, and ∇κ is the gradient vector. In general, $J^{\#}\dot{X}$ will utilize the needed joint motion to move the end-effector along the desired Cartesian trajectory, and the rest of the equation will move the joints in the null space to fulfill the optimization criteria. The details will vary between the different works, but the main focus of the gradient projection method is to define a ∇κ (and a factor λ occasionally) that creates an anthropomorphic motion. The benefit of this approach is that the end-effector will follow the defined trajectory regardless of the criteria used. It is also computationally efficient and can be used for real-time motion resolution. The drawback is that it resolves the inverse kinematics locally, i.e., it finds the motion that satisfies the constraints in the next step rather than a total motion profile that minimizes (or maximizes) the criteria for the whole trajectory. If the criterion is not about to be violated, then the result is the least-norm solution. The research that has been done in this field focuses on identifying the proper criteria that will lead to a biomimetic motion.

For instance, Zhao et al. [78] proposed a criterion that minimized the potential energy and factors in wrist discomfort to synthesize the motion. They were projected onto the null space to generate human motion. Alibeigi et al. [30] used the gradient of two cost functions to map human motions to an anthropomorphic robot (NAO) in real time. This allowed them to preserve the end-effector and joint configuration between the human and robot. Lura et al. [79] used a probability density function of the MoCap data to identify the human-like postures during certain tasks for use by prosthesis users. The optimization of the density function was conducted using the different increments of the workspace and data sizes. It was found that a trade-off between the accuracy and robustness existed. Poignant et al. [80] used the Rapid Upper Limb Assessment (RULA) to generate an ergonomically comfortable posture for prosthesis users. Xie et al. [81] used a minimum jerk criterion and defined a target arm pose to establish the desirable motion. while Artemiadis et al. [69] applied a probability distribution function. Using a Bayesian network for recording the motions in the joint, the authors were able to define the intra-dependency of the joints, which led to an objective function projected onto the null space for the joint. The other methods used more specific criteria for the gradient projection, such as the manipulability. In addition to measuring the comfort level a posture provides [82], the manipulability can also be used to simulate human movements. Gams et al. [48] used a manipulability maximization criterion to ensure that the arm maintained high levels of dexterity throughout the posture. A weighting matrix was developed through trial and error. A more advanced concept manipulability criterion was employed by Jaquier et al. [83]. In that work, a profile of the manipulability ellipsoid was used to control a humanoid robot. Building on that method, Jaquier et al. later showed [84] that the manipulability ellipsoid was task-dependent, and it could be transferred to robots for human-like movement. Sentis et al. [85] showed that it was possible to use the gradient projection to apply multiple criteria recursively to refine the performed motion.

### 4.3. Weighted Least-Norm Solution

The weighted least-norm (WLN) solution is a method similar to the gradient projection. The main difference is that it does not separate the rank and null spaces and has the general form shown in Equation (2).

(2) $$\dot{q}=WJ^{T}{\left(JWJ^{T}\right)}^{-1}\dot{X}$$

where W is a usually diagonal, weighting matrix, and $J^{T}$ is the transpose of the Jacobian matrix. Interestingly, this was the first equation used to resolve a redundant manipulator’s kinematics. In 1969 [86], Whitney proposed the WLN as a way to resolve the motion for both manipulators and human prosthetic devices (the gradient projection was proposed in 1977 by Alain Liegeois [87]). The WLN applied weighting factors on each joint that resolved the full motion (combined rank and null motion). Chan and Dubey [88] used this method to avoid a robot’s joint limits. In their paper, the comparison between the WLN and the gradient projection showed that the WLN always moved the mechanism away from the joint limits. In contrast, the gradient projection increased the compensation only when the limits were approached. Basically, the WLN was always active while the gradient projection was switched ‘on’ and ‘off’, leading to an increased compensation. In practice, the differences between these two methods should be minimal since biomimetic motion must always satisfy specific criteria, meaning that both methods will be active at all times.

Lura et al. [51] used optimization to extract the weights for able-bodied individuals and the incorporated joint limits to obtain a more personalized motion profile. Later, Menychtas et al. [50] extended that work to prosthesis users. While the initial results looked promising, the different anthropometrics of the prosthesis users couldn’t be generalized for use in a larger population. The most reasonable approach proved to be the use of the average joint velocity during a task [49]. In fact, the motion that was created did not have any statistically significant difference from the optimized weights. Another work that used the WLN was Gams et al. [48], which combined the gradient projection and the WLN to keep the two proposed criteria separate.

Ultimately, the gradient projection and the weighted least-norm share the same concept for resolving motion. The end-effector reaches a desired location, and the null space is controlled in a desirable manner to create a human-like motion. There is no consensus regarding which criterion can create the most human-like posture, so different researchers have attempted various constraints to fit their scope to achieve the best possible result. This will hold true in the following methods where more abstract criteria were used.

### 4.4. Constrained Optimization

Constrained optimization is the most popular method for solving the human motion inverse kinematics. The joint angles are selected as the variables of an objective function, and the motion profile derived from those characteristics is used to solve the problem. These characteristics can vary, but they are similar to the previous methods in that they reduce the solution space of a given position of an end-effector through a criterion. Constrained optimization has the characteristic (not necessarily a limitation) of mimicking a task that depends on several components, which may have a varied impact across tasks. In essence, this method clusters criteria without attempting to define the most important criterion. For example, Kusters and Glelen [89] found that the kinematics and dynamics affected motion differently depending on the task complexity. This means that the task would determine whether it should be resolved kinematically or dynamically. Most researchers choose their constraints based on a specific scope, which may result in more abstract criteria for the optimization. This contrasts with the previous methods where the criteria were chosen to be, if not intuitive, at least well-defined.

Lamperti et al. [73] used OpenSim to simulate dual-arm human motions and optimized the movement for an anthropomorphic robot using the same technique in order to reduce muscular fatigue. The researchers did not compare their results to the MoCap recordings, but instead, applied them to an open platform. Liarokapis et al. [25] defined their objective function based on three different geometric metrics: the distance between the human and the robot, the triangles’ area around the joints, and the convex hull. These metrics quantified the differences between the humans and robots so that the algorithm could map the anthropomorphic motion onto the mechanism. Gielniak et al. [35] used spatiotemporal coordination to produce human motion. Their method also added a variance to avoid non-anthropomorphic repeatability. They also experimented with more constraints and found that as the number of criteria increased, the motion became less human-like. Tomić et al. [29] worked on an algorithm to imitate human motion as well. Their optimization algorithm showed the limited error between the desired and robot joint angles. Similar to Liarokapis, their method required a hand trajectory. Kashima and Hori [90] explored how to create this trajectory and motion by applying a minimum angular jerk criterion. As a result of the optimization of the joint angles in the created path, it should be noted that the minimum jerk is one of the earliest criteria to create human-like trajectories, and it can be used in simple movements as well. The minimum angular jerk was also used to determine motion by Silva et al. [40], and their algorithm also considered the obstacle avoidance and joint limits. Albrecht et al. [28], employed a new approach by identifying a cost function extracted from a human motion capture system. The cost function was applied to a robot, which resulted in a biomimetic motion that respected the limitations of the robotic mechanism. Ishida et al. [91] used time-varying weights to optimize a composite cost function. This allowed them to mimic human motion, but the cost function applied soft constraints that could be violated at certain points. To achieve human-like synergies between two arms, Garcia et al. [33] mapped MoCap data onto a robot to generate the trajectories. Similarly, Choi et al. [92] used a constrained optimization to target human motion on multiple humanoid robots. Kim et al. [70] used a method of imitating human motion in which the swivel angle and a motion plane were defined to map the MoCap data onto an anthropomorphic robot. The MoCap was scaled accordingly to match the mechanism’s capabilities. Rosado et al. [93] also dealt with a similar problem. However, their solution involved feeding the human motions directly into the mechanism through a depth camera (KINECT). In order to refine the data collected from the sensor, they performed a constrained optimization. Once the higher-quality joint angle data were obtained, they were mapped directly onto the robot. The purpose of this work was to transfer skills from humans to robots.

A significant amount of work has been done using constrained optimization techniques for wearable robots and simulations of the human body. Kim et al. [94] calculated the swivel angle kinematically and dynamically, then synthesized the motion. They found that the results outperformed the output of a single criterion. The same was true of Kashi et al. [59], who used a minimal angular displacement criterion and a joint range availability criterion to calculate a human-like posture. In their work, the synthesis of the two components led to a better performance. A study by Vetter et al. [95] proposed two criteria to explain human motion. According to one postural model, the joint configuration depends purely on the end-effector’s position. In contrast, the previous posture influences the final joint posture in a transport model. In a similar vein, the work of Sha et al. [16] used multiple criteria to achieve a biomimetic solution. They used the total joint power, a center of mass displacement, and minimizing errors on the end-effector. It should be noted that they used a virtual full body model and not an anthropomorphic robot, which means that the mechanism was not limited. Fox et al. [58] proposed an optimization to generate human motion but did not map it on a robot. Their study aimed to examine how the shoulder will function after an operation (glenohumeral capsulorrhaphy—a surgery in which the soft tissue of the shoulder is repaired) and how the kinematics and muscle effort will change following the operation. Instead of creating a biomimetic motion profile for a robot, the purpose was to study the human body through simulation. Similarly, Al Borno et al. [96] used OpenSim to study the speed–accuracy trade-off. They found that this trade-off appeared due to greater task demands rather than signal-dependent noise. They also studied muscle synergies using simulation [44]. Along the same lines, Khatib et al. [97] accounted for the cross-joint coupling using robotic task prioritization techniques, and De Sapio et al. [46] utilized shoulder modeling to account for the cross-joint coupling.

### 4.5. Machine Learning, Neural Networks, and Reinforcement Learning

Machine learning (ML), neural networks (NN), and reinforcement learning (RL) methods are used to analyze the MoCap data using statistical methods to create the biomimetic motion. The data are used as the input and a pattern recognition takes place. As an output, the features that the algorithm deemed most relevant are extracted. The level of abstraction, in this case, is relatively high. Nevertheless, this does not mean that the criteria are treated as a black box; it simply means that the correlation between the features and the motion is more important than the specific way they affect the motion. ML is a massive field of research on its own, but in this section, the focus will be on the work that has been done in biomimetics. The main driving force of ML control is learning by demonstration. Therefore, various algorithms are used to extract useful information about motion [98]. An ML algorithm can transfer human motions to an anthropomorphic robot, allowing it to imitate, learn, and reproduce those motions. While ML methods can effectively mimic human motion from a large dataset, they do not focus on creating a biologically inspired criterion, unlike reinforcement learning. As a result, the imitated motions do not contain any inherent biological information and are not suitable for biomechanical simulations. Fang et al. [99] provided a survey on learning from imitation, but it mostly concerned transferring skills from experts to robots, not biomimicry. However, ML is able to mimic human motion efficiently and handle more complex tasks than other methods. In this paper, the scope is limited to the ML algorithms that are tested to human-like kinematics, rather than the much larger field of ML robot redundancy resolution. It is essential to keep in mind that the increased complexity of the involved algorithms does not necessarily guarantee a better human imitation. The most appropriate methodology will depend, as it has been stated already, on the specific task and the features that are relevant. For example, Zanchettin et al. [72] found a correlation between the angle of the swivel and the hand’s posture, which helped solve the problem of motion. Unlike the methods discussed in the section on closed-form solutions, this relationship allowed for the creation of a cluster of multiple solutions and then determined which solution would be the most appropriate using a multivariate correlation rather than limiting the number of available valid solutions to the problem. This article was notable because it correctly interpreted the human-like pattern as a synergistic behavior, which was not a common approach. Similarly, Artemiadis et al. [69] used a Bayesian network to determine the intra-joint connections and mapped out the motions using gradient projections. In contrast, Park et al. [100] used a set of MoCap data to construct a database of motion primitives. Their algorithm allowed them to derive a large set of efficient motions, but they also reported stability problems and joint limits. Wei et al. [101] tested a Bayesian network and a coupled NN (CNN) to create motion primitives and combined them with the projected human motion on anthropomorphic robots. They did not report any issues with the stability, but the CNN performed better than the Bayesian model. Su at al. [67] developed a nonlinear regression method that used an NN to control a teleoperated robot. They used the swivel angle to achieve a human-like motion in an industrial robot. To improve the robustness of the algorithm, they later employed a deep convolutional neural network [102]. Later, Su et al. [103] applied their methods to transfer skills from humans to manipulators. Chen et al. [32] also used NNs, but their approach involved reducing the dimensionality of the solution space in order to resolve the redundancy and limit the number of possible solutions. This resulted in a human-like motion and reduced the computational and memory costs. However, Elbasiony et al. [31] used the KINECT to demonstrate motions to a robot. Wen et al. [104] also used the KINECT to record motions that were used as the input to a multi-layer perception (MLP) NN to transfer biomimetic movements to a robot. Kratzer et al. [105] used a rather interesting approach. In their work, they used a recurrent NN to plan motions offline and gradient-based trajectory optimization online to account for the obstacles and other external parameters.

RL involves evaluating a cost function iteratively and refining its parameters with each iteration. This method avoids complex inverse kinematic calculations and allows the robot to learn autonomously by exploring the solution space until the cost function converges. The main difference compared to other ML algorithms is that the system doesn’t explicitly try to imitate the given dataset to the best of its ability. Instead, it tries to explore the solution space to find allowable and ideally optimal solutions. Xie et al. [106] provided more details by focusing on path planning. As a result, researchers can experiment with bio-inspired criteria that aim to recreate the biological functions that create motion with reasonable assumptions. It allows for researchers to experiment with the cost function, and the final algorithm will be a combination of the initial criteria and the motions that the robot is capable of and allowed to produce. In order to solve the cost function, it is necessary to find a balance between exploring the solution space and utilizing the current knowledge available at that time. As a result, the robot is simply following the given information and it gains a considerable amount of new knowledge due to the exploration–exploitation trade-off by resolving the kinematics.

A collision-free, energy-saving trajectory can be found using the dynamic movement primitives by Liu et al. [107]. Based on this, the researchers proposed an adaptive exploration strategy, which allowed the robot to fine-tune the noise decay parameters, thus helping the algorithm arrive at a solution faster. A method was presented by Guenter et al. [108] that used demonstration to teach a robot, as well as reinforcement learning to enable the robot to find a different solution when unexpected perturbations arose. They used a Gaussian mixture model (GMM) to find an alternative solution. The same approach was used by Calinon and Bilard [27] when they taught a robot using a human in the loop and encoded the motion of the human with the GMM. To transfer the gestures from the human to the anthropomorphic robot, the movements were first demonstrated using MoCap, and then the robot’s performance was manually adjusted. This resulted in successfully transferring the movement from the human to the robot. Billard et al. [57] performed reinforced learning with the use of neural networks (NN). They used a hierarchy of NNs that imitated some parts of the brain that are responsible for motor control. The motions were demonstrated to the system. Since the NNs were modeled after the brain, the system would reiterate the observed motions internally to generate their own configurations; a process that is similar to biological learning by seeing and observing. This method is different than the NN methods discussed in the next section because the network was used to reinforce a behavior and not to statistically determine a pattern. Lim et al. [109] used principal component analysis (PCA) to decompose the motions into motion primitives, which the robot could then combine to achieve fast and efficient motion similar to that of a human. Jenkins et al. [110] employed a bio-inspired classification of percepto-motor primitives to generate human-like motion from these primitives. Yang et al. [111] developed reward functions using MoCap, a deep deterministic policy gradient (DDPG), and hindsight experience replay (HER) for the first time, enabling the robot to mimic human motions. Fan et al. [43] used reinforcement learning to control an anthropomorphic robot using artificial muscles. However, their work differed in the sense that the system had to learn how to activate the muscles in order to move the robot.

## 5. Discussion and Future Works

In this paper the progress that has been achieved in biomimetic robotic was presented. It is essential to note that the scope was not to simply catalogue the multiple ways that redundant robots can be controlled but to focus on the interconnection between biomimetics and biomechanics. Indeed, some of the more relevant applications were the ones that applied motion control algorithms to study the human body. In order to go a step further, a good understanding of the human body is required. It is worth noting that the study of human motion is an active field of research, and not all aspects of it are relevant to robotics. Despite the similarities between biomechanics and robot control, certain aspects must be taken into consideration.

The first consideration is the modeling of the human body. Skeletal joints modeling is a field of study on its own. This is because every joint exhibits a rotation and translation in all dimensions, though not all DOFs are as important or even voluntary. For example, the carrying angle of the arm is perpendicular to the elbow’s flexion/extension axis but it changes only proportionally to the elbow’s motion since there is no combination of muscles that can control it independently. Another issue regarding the modeling of the human anatomy is that some joints do not have a well-defined joint center. For example, the wrist rotation is produced when the ulna and radius bones go from being parallel to crossing over each other. This raises the question of where the center of rotation should be placed. Similarly, the shoulder joint can be challenging to model; the clavicle, scapula, and humerus form a complex joint that produces a rotational motion that appears to have its joint center move. The hip and the knee joint centers can also be challenging to define due to their large range of motion that can exhibit a translation at certain angles. This makes the modeling of the body’s major joints difficult. In general, the International Society of Biomechanics (ISB) recommendations are a good starting point [112], but every researcher will adjust them based on their scope. A de facto standardization is used by most researchers, with the lower limb being considered the most well-standardized, but even that can vary considerably [113]. In the scope of biomimetics, the question of the joint center’s placement affects the design of the anthropomorphic robot. For example, the NAO (Aldebaran, Paris, France) uses five DOFs on the arm, and the LBR ii (KUKA, Augsburg, Germany) system, used in many works reviewed here, has seven DOFs. The ISB recommendations [114] proposed 11 DOFs that start at the sternoclavicular joint, with many researchers using their own variation [60,115,116,117]. More DOFs will not necessarily increase the accuracy of the mimicked motion, but they will increase the control algorithm’s complexity. Similar to biomimetics, the scope of the application will be a significant factor for deciding the number of DOFs.

Considering the problem of human DOFs and their control, Latash [118] proposed that the problem should be seen as a matter of motor abundance rather than redundancy. According to motor abundance, the central nervous system (CNS) does not micromanage every joint but provides a generic motion strategy. In this manner, Scholz and Schöner [119] determined that the precise motion pattern should be dependent on the current state of the system. The main observation was that humans showed a variation in their joint angles during repeating motions, but the variation did not affect the task. In light of this, they divided the joint angle differences into those that did not affect the performed task (good variation), and those that affected the task (bad variation). The motions that positively affected the task were considered to lie on the uncontrolled manifold (UCM) of the solution space, and therefore, there was no need for the CNS to control them directly, or at least precisely. The bad variation existed on a space perpendicular to the UCM. It was easy to see the UCM motions as the null space and the rank space variation as the perpendicular ‘bad variation’ space that affected the task if it was not controlled properly.

The primary method for performing the UCM analyses was to construct a geometric model of the body, calculate the Jacobian of the model, and then explore the null space of the model [120]. The research demonstrated that the joint angles of the arm could change with multiple repetitions of the same task, but a controlled variable, such as the end-effector pose or the total force applied when gripping, would remain unchanged. The first experiment that reported on the joint variability that didn’t seem to affect the hand trajectory was that of Bernstein [121]. He instructed blacksmiths to use a hammer to hit a spot while he recorded their motions. Even though they all managed to hit the same spot consistently, their joint configuration varied between each repetition. The research that has been conducted since Bernstein’s experiment shed further light into this apparent paradox of consistent trajectories with inconsistent joint angles. The controlled variable (the hand holding the hammer) was not affected because the change in the joint angles produced synergies that kept the hand stable and consistent despite the variation in the joint angles. In simple words, the change in one joint was offset by a change in another joint, leading to a stable trajectory. The UCM assumes that the state of the system will determine the final joint configuration rather than an extremely dense signal from the CNS that will micromanage every joint with absolute accuracy. In essence, the human body doesn’t reject certain solutions but chooses the one that is more accessible at that specific instance. As far as biomimetics are concerned, the concept of searching for one unique, repeatable solution by adding specific criteria might need to be revised, considering that even the human body does not recreate its own solutions. Finally, even though it’s outside the scope of this review, research has already shown the importance of passive dynamics and chaos theory on walking robots [122]. Though there hasn’t been as much research for the upper body, it is worth considering how chaos theory and passive dynamics could be responsible for the documented variability on upper extremity motions.

Future research should prioritize the resolution of the fragmented framework that currently exists in both the biomechanical analyses and biomimetic robots. Specifically, it is essential to achieve consistency in the mathematical description of the motion between robots and the human body, enabling robots to serve as a potent testbed to understand the CNS. While analytical approaches have attempted to explain the CNS, ML methods have proven more powerful, despite their limited ability, in highlighting specific biological aspects. Thus, future work could focus on improving and explaining the ability of the ML results.

To achieve a clear consensus on the kinematic structure of the human body, it is necessary to propose specific criteria that a robot needs to follow to be considered biomimetic. Furthermore, learning through imitation algorithms, which aim to control a robot in a specific environment rather than explicitly mimicking human motion, should clearly identify the assumptions that do not occur in the human body, such as the limited DOFs or completely different dynamics, to distinguish the methods that are not viable for biomimicry but are suitable for bio-inspired industrial robots. In general, it is necessary to reframe the concept of biomimetics to avoid confusion with the other fields of robotics. This is becoming increasingly important as more advanced ML algorithms are introduced, and vast libraries of human motion recordings are accumulated to solve complex and specialized problems.

## 6. Conclusions

A wide variety of techniques can be used to solve the inverse kinematics of the human body to drive an anthropomorphic robot in many different ways. The scope of the application determines the methods and the level of acceptable mimicry. However, there must be a definitive method for reconstructing human motion and implementing it as a function. There is a great deal of research that needs to be done in order to gain an understanding of how the CNS affects a person’s movements.

Despite the increasing prevalence of redundant robot research, a formal structure is needed to organize the experimental setups and optimize the approaches for various biomimetic experiments. By categorizing the various data collection methods, robot manipulators, experimental setups, and optimization techniques, this review contributed to a structured approach. First, we categorized the human motor control into two fundamental sections. In the first section, different experimental setups were used. We categorized this section according to the different models, separating it into simulation- and lab-based setups. As part of the second section, we examined the various optimization methods. Within this section, we divided them into different categories: machine learning/neural networks, reinforcement learning, weighted least-norm, constrained optimization, and gradient projection.

Biomechanics research has achieved significant advances, but it needs to further interact with biomimetic research to develop simulation tools that can be used to examine and recreate human motion. Although neural networks and machine learning have been proven to be successful methods for biomimetics, their efficiency still needs to be proven for novel trajectory applications. Researchers should focus on learning from human demonstrations to improve the robustness of mimicking to better understand the various performance criteria, gravitational torque optimization, manipulability, velocity ratio, and muscle effort that humans subconsciously optimize when performing a particular task. A subtle but critical issue that was observed during this review was that each method was separate from the other methods. Researchers developed their own methods that cannot be transferred to augment already existing work. To tackle this, a set of standardized modular frameworks based on a large dataset, optimization methods, and experimental approaches would be needed to facilitate the development of a new research platform. This will allow researchers to build on previous studies and revisit certain concepts without the need to recreate studies that have already been developed with mixed results.

## Figures and Tables

**Table 1:** Key Features of the Various Types of Motion Capture Systems.

Technology	Advantages	Disadvantages
Optical tracking system using passive markers	Precision < 1 mm.	Occlusion.
	Reflective markers without any wires or battery packs.	Post-processing expectancy.
	Reliable and flexible sensor arrangement.	Not for outdoor use.
Optical tracking system using active markers	Precision < 1 mm.	Occlusion.
		Post-processing expectancy.
	Higher precision compared to passive markers.	Presence of wires and battery packs makes it cumbersome.
		Not for outdoor use.
Optical tracking using a markerless vision-based system	Wireless.	Occlusion.
	Moderate reliability.	High noise.
	Can be used for indoor outdoor setup.	
	Without any markers, wires, or battery packs.	Sensitive to lighting.
	Contextual information.	
Inertial sensors tracking system	Wireless.	Sensitive to magnetic field.
	Precision < 1 mm with fast calibration.	Post-processing expectancy.
	Can be used for indoor outdoor setup.	High noise.
Magnetic marker tracking system	Wireless.	Sensitive to magnetic field.
	Moderate reliability.	Post-processing expectancy.
	Can be used for indoor outdoor setup.	High noise.
		Limited range.

## Data Availability

The data utilized in this review article are available in the sources cited and can be accessed through web searches or by contacting the authors of the original studies.
